# Emergency Presentations of Meckel's Diverticulum in Adults

**DOI:** 10.1155/2022/6912043

**Published:** 2022-08-25

**Authors:** Ayad Ahmad Mohammed, Mohammed Rasheed Mohammed

**Affiliations:** Department of Surgery, College of Medicine, University of Duhok, Kurdistan Region, DUHOK, Iraq

## Abstract

**Introduction:**

Meckel's diverticulum is the commonest congenital anomaly of the gastrointestinal tract in humans that is commonly encountered during surgical practice as the cause of the patient's presentation or as an incidental finding during other unrelated procedures. Most clinical symptoms are caused due to its complications.

**Results:**

The mean age of the involved patients was 24.79 years with slight male predominance, 62.9% males compared to 37.1% females. The mean length of the diverticulum was 55.21 cm. The most common emergency presentation was right lower quadrant abdominal pain in 31% of the patients, intestinal obstruction in 28.6%, acute lower abdominal pain and guarding and acute abdomen in 18.6% and 15.7% of patients, respectively, bleeding per rectum in 2.9%, acute right upper quadrant abdominal pain in 1.4%, and obstructed paraumbilical hernia containing the diverticulum in one patient. Perforation of the Meckel's diverticulum was reported in 18.6%. Histopathological examination showed acute inflammation in the wall of the diverticulum in 37.1%, lymphoid hyperplasia in 24.3%, hemorrhagic necrosis in 22.9%, and chronic inflammation in 8.6%. Ectopic mucosa was detected in 50% of the cases, gastric mucosa was detected in 42.86%, ectopic pancreatic mucosa was detected in 5.71%, and both gastric and pancreatic types in 1.43%.

**Conclusion:**

Long diverticula are more liable to develop complications. At surgery, inspection and palpation of the wall of the diverticulum must be done for any evidence of inflammation, necrosis, perforation, or abnormal thickening of the walls of the diverticulum. Resection of the segment of the bowel that contains the diverticulum with primary anastomosis is preferable to other procedures due to the risk of leaving behind an abnormal heterotopic mucosa.

## 1. Introduction

Meckel's diverticulum is commonly encountered during surgical practice as the cause of the patient's presentation or as an incidental finding during other unrelated procedures. It was described first in 1598 by Fabricius Hildanus, but its name is derived from a German anatomist known as Johann Friedrich Meckel who described both embryological and pathological features of the diverticulum in 1809. It generally remains silent but some life-threatening complications may occur thus increasing its importance in clinical practice. A detailed knowledge about the anatomical and pathophysiological properties of the Meckel's diverticulum is required to deal with such presentations. [1].

The yolk sac of the developing embryo is connected to the primitive gut by the yolk stalk or vitelline (i.e., Omphalo-mesenteric) duct. Meckel's diverticulum represents the remnant of the vitellointestinal duct (prenatal yolkstalk). This duct normally regresses in the period between the fifth and seventh weeks of fetal life. Various anomalies may be seen when there is failure of this process of regression, including the presence of a Meckel's diverticulum, a fibrous cord attaching the distal ileum to the abdominal wall, an umbilical-intestinal fistula, a cyst lined by mucosa, or an umbilical sinus. [2].

Meckel's diverticulum is the commonest congenital anomaly of the gastrointestinal tract in humans and is present in approximately 2–3% of the population. It is located on the antimesenteric border of the ileum approximately 45–60 cm proximal to the ileocecal junction and ranged from 3–5 cm in most of the patients. [3].

It is a true diverticulum; i.e., the walls contain all the three layers of the intestinal wall and it has its own blood supply arising from the superior mesenteric artery. Because the cells lining the vitelline duct are pluripotent cells, the mucosa of the diverticulum may contain heterotopic gastric mucosa (50%), pancreatic mucosa (5%), and less commonly colonic mucosa, endometriosis, or hepato-biliary tissue. These types of mucosae make it vulnerable to other complications such as hemorrhage, chronic peptic ulceration, and perforation. [4].

Clinical symptoms are caused by complications like peptic ulceration with bleeding; Meckel's diverticulitis; intestinal obstruction, intussusception, volvulus, on some rare occasions the diverticulum enters a hernia sac; and the development of cancer within the diverticulum which has been reported in some cases. Although most complications manifest during the childhood period, but they may be present in adult life as well. [3].

The preoperative diagnosis of a complicated Meckel's diverticulum can be challenging and is often difficult to establish. The challenge is especially true in many adult patients because the clinical symptoms and imaging features of a complicated Meckel diverticulum overlap with those of many other disorders that cause acute abdominal pain or gastrointestinal bleeding.

The purpose of this article is to review the embryologic, clinical, pathologic, and radiologic features of Meckel diverticulum with an emphasis on radiologic-pathologic correlation.

### 1.1. Patients and Methods

This retrospective descriptive study included 70 patients who presented to Duhok Emergency Hospital complaining of acute abdomen or clinical suspicion of acute appendicitis and were operated on, and during surgery they were diagnosed as complicated Meckel's diverticulum. Some patients who were presented with massive lower GIT bleeding that was not responding to other forms of treatment and underwent surgery for that, and the cause was attributed to Meckel's diverticulum during surgery, were also included in this study. The Meckel's diverticulum with the associated other samples such as the appendix were removed and sent for histopathological analysis. Patients with incomplete data were excluded from this study. Patients with ages less than 18 years and those who refused to be enrolled were also excluded.

### 1.2. Statistical Analyses

The descriptive purpose of this study is to describe the different emergency clinical presentations and histopathological analyses of patients with Meckel's diverticulum.

Data are displayed in terms of frequency, mean, median, and standard deviations. The statistical calculations were done using the Statistical Package for Social Sciences (SPSS 25 : 00 IBM : USA).

An ethical committee agreement is granted from the scientific committee at the College of Medicine, Duhok University.

## 2. Results

**Figure 1:**
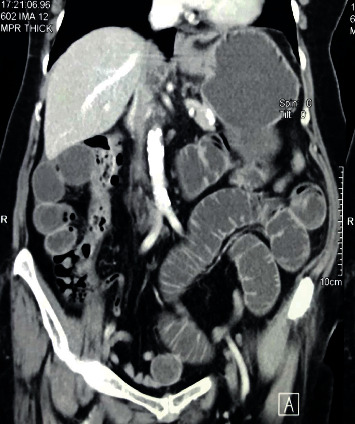
CT scan of the abdomen showing intestinal obstruction caused by Meckel's diverticulum.

The majority of the patients in our study were males, and the mean age of those was 24.79 years (SD 12.239). The most common clinical presentation was right lower quadrant acute abdominal pain in 31.4%, followed by intestinal obstruction and lower abdominal pain in 28.6% and 18.6%, respectively.  Table 1 & Figure 1.

The mean length of the Meckel's diverticulum was 55.21 mm and the mean width was 19.67 mm.  Table 2.

The most common intraoperative findings were signs of inflammation in both appendix and the Meckel's diverticulum (41.4%), followed by finding of a perforated Meckel's diverticulum (18.6%), and intestinal obstruction caused by adhesions due to the diverticulum (15.7%). Other findings are shown in Table 3.

The most common histopathological findings were acute inflammation and lymphoid hyperplasia in the walls of the diverticulum in 37.1% and 24.3%, respectively, other findings are described in Table 4.

**Figure 2:**
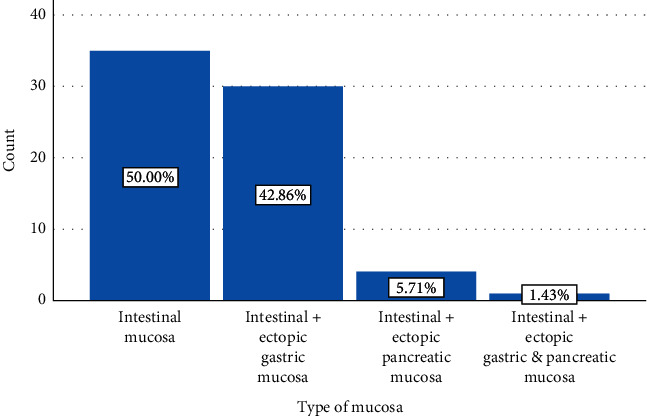
A simple bar chart showing the types of the mucosal linings of the Meckel's diverticulum.

**Figure 3:**
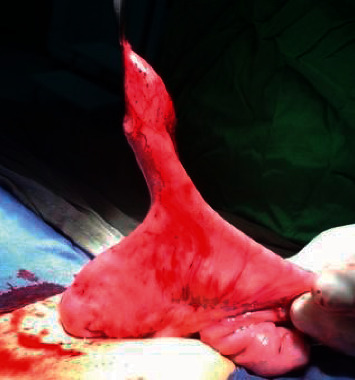
An intraoperative picture showing a Meckel's diverticulum that caused bleeding per rectum and the histopathology showed the presence of an ectopic gastric mucosa.

The most common types of the mucosal lining was the intestinal mucosa with no ectopic other types of mucosae in 35 patients, ectopic gastric mucosa was detected in 30 patients, ectopic pancreatic mucosa was detected in 4 patients and the ectopic both intestinal and pancreatic mucosae were detected in 1 patient Figures 2 and 3.

## 3. Discussion

Meckel's diverticulum is the most common congenital malformation of the GIT with around 2% population being affected. The lifetime risk for the development of complications related to the diverticulum is estimated to be around 4%. The probability for the development of complications decreases with age. [5].

The diagnosis in adults is mostly incidental during other procedures or when it causes complications and emergency presentations. The treatment of incidentally discovered diverticula remains controversial, while the treatment of symptomatic ones requires definitive surgical intervention. According to large series of patients' literature, surgery is not indicated in the absence of risk factors for complications such as male gender, age smaller than 40 years, the length of the diverticulum greater than 2 cm, and the presence of an ectopic mucosa. In our study, the mean length of the diverticulum was 55.21 cm (SD 26.37), supporting the fact that longer diverticula are more liable to develop complications. [4–7].

The mean age of the involved patients in our study was 24.79 years (SD 12.239), with slight male predominance, 62.9% males compared to 37.1% females. Studies and reviews document a larger incidence in males and regard males as a risk factor for the development of complications. [5].

In this particular study, we studied the emergency presentations of the diverticulum in which all of the involved patients required emergency surgeries. The most common emergency presentation in our study was right lower quadrant abdominal pain in 31% of the patients (22 patients). Intestinal obstruction was the second most common emergency presentation in 28.6% of them (20 patients), followed by acute lower abdominal pain and guarding and acute abdomen in 18.6% and 15.7% of patients, respectively. Other types of presentations were less common such as bleeding per rectum (2.9%), acute right upper quadrant abdominal pain (1.4%), and obstructed paraumbilical hernia containing the diverticulum in one patient. The type of surgical intervention include excision of the diverticulum, or wedge resection, segmental resection. The selection of the procedure depends on the type of the presentation, the integrity of the base of the diverticulum and the adjacent ileum, and the presence of ectopic mucosa within the diverticulum. Some authors document that resection of the segment of the bowel that contains the diverticulum followed by primary anastomosis is preferable to wedge resection or tangential mechanical stapling due to the risk of leaving behind an abnormal heterotopic mucosa. [4, 5, 8, 9].

At surgery, inspection and palpation of the wall of the diverticulum must be done for any evidence of inflammation, necrosis, perforation, or abnormal thickening of the walls of the diverticulum. Searching for other pathology is also part of the operative procedure. As most patients presented with right lower quadrant abdominal pain, appendectomy was performed as part of the procedure. Intestinal obstruction was diagnosed in 28.6% of patients. Intestinal obstruction is caused either by adhesion bands with the diverticulum, intussusception, volvulus, or perforation. Cases of hernia containing Meckel diverticulum are rare. We encountered one patient with an obstructed paraumbilical hernia containing the diverticulum, and cases of internal hernia or inguinal hernia containing the diverticulum have been reported. The latter is termed Littre's hernia. [10–15].

In addition to resection of the diverticulum, relieving the intestinal obstruction, dealing with intraperitoneal sepsis, reduction of intussusception, and dealing with any other findings from surgery should be done. Perforation of the Meckel's diverticulum was reported in 18.6% of our patients, and those patients were treated with segmental resection. Some authors recommend a laparoscopic approach for patients that have a history of abdominal pain mimicking acute appendicitis, a history of bloody stools, or chronic recurrent abdominal pain. However in all of our patients, we adopted the conventional open approach. [4, 16].

Bleeding per rectum due to MD was seen in 2.9% of our patients and in all of them was so severe that the available modalities of diagnosis and treatment failed to stop it and mandated an emergency intervention. The best option for the diagnosis of bleeding from the diverticulum is balloon assisted enteroscopy that provides the highest diagnostic accuracy. Other modalities for diagnosis of bleeding from the diverticulum include Meckel scan, capsule endoscopy, angiography, CT-scan, and small bowel follow through. There are no clear guidelines regarding the indication of surgery for patients with lower GIT hemorrhage, but the most common indication is for patients with hemodynamic instability on arrival to the emergency department. [17, 18].

All of the resected samples were sent for histopathological examination, which showed acute inflammation in the wall of the diverticulum in 37.1% (26 patients), lymphoid hyperplasia in 24.3% (17 patients), hemorrhagic necrosis in 22.9% (16 patients), and chronic inflammation in 8.6% (6 patients). The presence of the ectopic mucosa cannot be predicted by the clinical presentation or the palpation of the walls of the diverticulum. The types of the mucosa can be exactly diagnosed only by histopathological examination. In our patients, the presence of ectopic mucosa was detected in 50% of the cases, the presence of ectopic gastric mucosa was detected in 42.86% of them, the presence of ectopic pancreatic mucosa was detected in 5.71% of patients, and the presence of both gastric and pancreatic types was detected in 1.43%. [4].

## 4. Conclusion

Long diverticula are more liable to develop complications. At surgery, inspection and palpation of the wall of the diverticulum must be done for any evidence of inflammation, necrosis, perforation, or abnormal thickening of the walls of the diverticulum. Resection of the segment of the bowel that contains the diverticulum with primary anastomosis is preferable to other procedures due to the risk of leaving behind an abnormal heterotopic mucosa.

## Figures and Tables

**Table 1:** Showing the characteristics of the participants and the various types of presentations.

Main category	Subcategories	Frequency	Percent
Age (M;SD)		24.79	12.239

Sex	Male	44	62.9
	Female	26	37.1

Presentation	Right lower quadrant abdominal pain	22	31.4
	Intestinal obstruction	20	28.6
	Lower abdominal pain	13	18.6
	Acute abdomen	11	15.7
	Bleeding per rectum	2	2.9
	Right hypochondrial pain	1	1.4
	Obstructed paraumbilical hernia	1	1.4

**Table 2:** Showing the dimensions of the Meckel's diverticulum.

Category	Range	Mean	Standard deviation
Length of diverticulum in millimeters	12–150	55.21	26.378
Width of the diverticulum in millimeters	5–40	19.67	7.974

**Table 3:** Showing the intraoperative findings.

Operative findings	Frequency	Percent
Appendectomy + excision of Meckel's diverticulum (signs of inflammation in both^*∗*^)	29	41.4
Perforation of Meckel's diverticulum	13	18.6
Intestinal obstruction due to adhesions caused by Meckel's diverticulum	11	15.7
Intussusception caused by Meckel's diverticulum	8	11.4
Excision of Meckel's diverticulum (signs of inflammation in Meckel's)	6	8.6
Phlegmonous mass formed by the inflamed Meckel's diverticulum and bowels	1	1.4
Obstructed paraumbilical hernia containing Meckel's diverticulum	1	1.4
Omphalo-mesenteric cyst	1	1.4

^*∗*^ *This include increased wall thickness and hyperemia compared to other bowel segments.*

**Table 4:** Showing the histopathological findings of the excised samples.

Histopathological findings	Frequency	Percent
Acute inflammation in the wall of Meckel's diverticulum	26	37.1
Lymphoid hyperplasia in the wall of Meckel's diverticulum	17	24.3
Hemorrhagic necrosis in the wall of Meckel's diverticulum	16	22.9
Normal Meckel's + inflamed appendix	2	2.9
Chronic inflammation + fibrosis in the wall of Meckel's diverticulum	6	8.6
Normal Meckel's diverticulum	2	2.9
Mucinous cystic tumor of the appendix + inflammation in the wall of Meckel's diverticulum	1	1.4

## Data Availability

Data will be available for the journal office when required.
